# Trends and Inequities Related to Antenatal Care Utilization Among Pakistani Women From 1990 to 2019: An Insight From Demographic and Health Surveys

**DOI:** 10.1155/jp/5592589

**Published:** 2026-07-08

**Authors:** Sarosh Iqbal, Rubeena Zakar, Muhammad Aftab, Naveeda Noreen, Nazoora Mnal Zakar, Shariq Ali Khan

**Affiliations:** ^1^ Department of Sociology, School of Social Sciences, Forman Christian College (A Chartered University), Lahore, Pakistan, fccollege.edu.pk; ^2^ Department of Gender Studies, University of the Punjab, Lahore, Pakistan, pu.edu.pk; ^3^ Office of Planning & Development, Lahore, Pakistan; ^4^ Independent Researcher, Lahore, Pakistan; ^5^ Akhtar Saeed Medical College, Lahore, Pakistan; ^6^ Institute of Health and Wellbeing, Federation University Australia, Melbourne, Victoria, Australia, federation.edu.au

**Keywords:** 1990–2019, antenatal care, inequities, married women, Pakistan, trend

## Abstract

**Background:**

Antenatal care (ANC) utilization is an essential component of the continuum of maternal healthcare services to promote positive health outcomes for both the mother and newborns. An adequate number of ANC visits (at least 4 visits or more) provides pregnant women with an optimistic pregnancy experience. Otherwise, the inadequacy of ANC may result in adverse health outcomes, such as the risk of preterm births, low birth weight, and perinatal mortality. This research is aimed at investigating trends and inequities related to ANC utilization among women of reproductive age (15–49 years) across Pakistan from 1990 to 2019, covering almost the last three decades.

**Methods:**

This study used secondary data for analysis of all rounds of Pakistan Demographic and Health Surveys (PDHSs) from 1990 to 2019, including the latest Pakistan Maternal Mortality Survey 2019. The analysis included those married women of reproductive age (MWRA) who had live births in the last 5 years preceding all waves of PDHSs. Women′s sample weights were applied, using the IBM SPSS Version 25. We carried out univariate descriptive analysis and bivariate cross‐tabulation analysis to determine the relationship between outcome and explanatory variables, where a *p* value ≤ 0.05 was considered statistically significant. Further, bivariate and multivariate logistic regressions were applied to explore the association of adequate ANC visits with explanatory variables, where odds ratios (ORs) and adjusted odds ratios (AORs) at 95% confidence intervals (CIs) were calculated.

**Results:**

The research witnessed an upward trend in the utilization of adequate ANC visits, with an increase from 14.2% to 54.5% from 1990 to 2019 in Pakistan. Results showed that adequate numbers of ANC utilization had a strong association with population characteristics of MWRAs aged 15–49 years. Particularly, it was found to be higher among 25–34‐year‐old women who attained higher education and were employed in professional/managerial occupations. However, a variation of inequities related to ANC utilization was also manifested from other key factors, such as poor wealth quintile, nonavailability of transportation, women′s and husbands′ lower education levels, lack of mass media exposure, women′s low autonomy, and shortage of healthcare providers (LHWs). Further, results also revealed a strong association between adequate ANC utilization and women′s need factors, such as parity, gravidity, and desire for more children.

**Conclusion:**

This research concludes the increased utilization of adequate ANC visits in Pakistan over the past three decades; nonetheless, persistent disparities necessitate immediate attention. Focused interventions, particularly in rural and remote areas, are imperative to increase the uptake of ANC and achieve universal healthcare for mothers and newborns. Addressing service delivery gaps and expanding the skilled healthcare workforce are pivotal for effective ANC services provision and promotion of its benefits. To further enhance ANC utilization, government efforts should prioritize investment in community awareness campaigns aimed at educating and empowering pregnant women and their families.

## 1. Introduction

Pregnancies represent a physiological condition characterized by the risk of complications, affecting the health of both the mother and her fetus [1]. A high maternal and infant mortality burden is predominant in lower‐middle‐income countries (LMICs), accounting for 94% of maternal deaths globally [2–4]. Among these, 68% of maternal deaths occur in sub‐Saharan Africa, followed by 19% in Southern Asia [3]. The literature informed that almost 75% of all maternal deaths would have been averted if mothers had received appropriate maternal healthcare services [5]. Evidence revealed that preventable maternal deaths are mainly attributed to a low level of maternal health service utilization, particularly belonging to the poorest segment of the population [6].

Antenatal care (ANC) utilization is an essential component of the continuum of maternal healthcare services to promote positive health outcomes for both the mother and her fetus [1]. ANC refers to the care given to pregnant women by a healthcare professional to monitor the well‐being of both the mother and her offspring. Effective ANC may decrease up to 50%–70% of maternal and infant deaths through timely clinical diagnosis, prevention of obstetric complications, providing health education about the management of high‐risk pregnancy, and promotion of skilled birth attendance [1, 7]. During ANC, priority areas of action also include maternal and fetal nutritional assessment, along with preventive measures and interventions for common physiological symptoms to achieve healthy motherhood [1].

The World Health Organization (WHO) recommends at least four ANC visits before childbirth to attain optimal healthy outcomes for pregnant women without any perinatal problems [1]. Subsequently, the four‐visit focused approach was replaced by the “2016 WHO ANC Model,” entailing comprehensive guidelines for routine management of pregnant women [1, 8]. This model emphasized that a pregnant woman should attend a minimum of eight ANC visits (i.e., first visit in the first trimester (up to 12 weeks of gestation), followed by two visits during second trimester (at 20 and 26 weeks of gestation), and subsequently five visits during third trimester [at 30, 34, 36, 38, and 40 weeks of pregnancy]) [8]. The WHO accentuated that an increased number of visits provides pregnant women with an optimistic pregnancy experience [1]. Otherwise, the inadequacy of ANC may result in adverse health outcomes, such as the risk of preterm births, low birth weight, and perinatal mortality [9, 10]. However, attending to the recommended ANC visits is only a proxy indicator of maternal health service provision as it disregards the quality of ANC [11]. Therefore, there are serious concerns about suboptimal ANC quality, driving persistently adverse maternal and newborn health outcomes across LMICs [12].

The developing country of Pakistan, with a population of 208 million, reported one of the highest maternal and infant mortality across the world. The recent estimates of country‐specific maternal mortality ratio (MMR) of 276 deaths per 100,000 live births and infant mortality rate (IMR) of 56.9 per 1000 live births, despite a substantial reduction, still lag in achieving the Sustainable Development Goals (SDGs) target by 2030 [13, 14]. Previous research informed that the availability of quality maternal healthcare services and utilization are inadequate in the country, where utilization of ANC with a single visit was recorded at 73%, followed by 52% skilled birth attendance during delivery [15]. Further, less than half of pregnant women in Pakistan receive ANC only from a qualified healthcare professional; however, a large proportion of women receive maternal care services from traditional birth attendants/DAIs [16, 17], raising concerns for maternal and infant mortality. Above all, the majority of women do not receive the necessary components of quality ANC services, which makes it more worrisome for both maternal and newborn health [18]. Moreover, the survival chance of maternal and neonatal mortality can be significantly reduced with improved ANC services [19].

Considering the critical maternal and infant mortality indicator for Pakistan, the optimal number of ANC visits may play a significant role in improving the health and well‐being of pregnant women. Obstetric complications and morbidities may be averted during ANC visits [20]. Particularly, the role of ANC is critical to educate pregnant women from all socioeconomic strata to avoid high‐risk pregnancies, debunk pregnancy‐related myths, and establish a liaison with health professionals for effective healthcare services [21, 22]. Pregnant women in the country can improve their own and their fetal health during pregnancy, delivery, and the postpartum period through continuous health education and monitoring of the warning signs and symptoms during ANC visits [23].

Altogether, the maternal, reproductive, and child health indicators show glaring disparities; therefore, Pakistan is ranked in the Top 10 with unequal healthcare interventions [24]. Particularly, the situation is worse for the women, who lack education, belong to the lowest quintile of income, and reside in rural areas [16]. A large strand of the literature revealed that ANC serves as a primary protective strategy for mothers′ and newborns′ survival at the local level, using WHO′s recommended ANC components, including measurements of blood pressure, examination of urine, vaccination against tetanus toxoid, proper intake of iron and folic acid supplements, and counseling regarding warning symptoms of pregnancy by a qualified healthcare professional [22, 25–27].

Given the significance of ANC, there is a gap in existing literature investigating trends of ANC utilization and inequities related to ANC at the country level, although several researches have been conducted in Pakistan, highlighting the role of ANC in maternal and newborn health [8, 16, 22–27].

Nonetheless, there is a paucity of research examining the trend of ANC utilization. Hence, this research is aimed at ascertaining the trend and inequities related to ANC utilization among women of reproductive age (15–49 years) across Pakistan from 1990 to 2019, covering almost three decades.

## 2. Conceptual Framework

**Figure 1:**
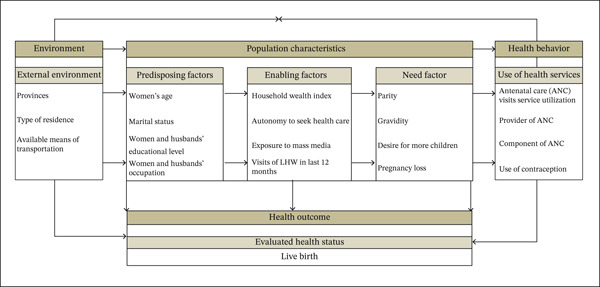
Conceptual framework for examining trends and inequities in ANC utilization in Pakistan.

Seeking guidance from Andersen′s behavioral model of health service utilization, this research is aimed at exploring the trend and inequities related to ANC utilization in Pakistan [28, 29]. This model has been used extensively to identify determinants and understand healthcare utilization in varied settings, including ANC [30–32]. This model facilitates the incorporation of multilevel individual and contextual determinants of healthcare use within three dimensions (i.e., external environmental factors related to the healthcare system; population characteristics, e.g., predisposing, enabling, and need‐based factors; health behaviors; and health outcomes) (Figure 1). This model was used a priori to guide variable selection and their grouping as independent variables.

## 3. Methods and Material

### 3.1. Data Source

This study used the secondary data for analysis of Pakistan Demographic and Health Surveys (PDHSs)—the nationally representative dataset of key health indicators. Overall, four cross‐sectional and independent rounds of PDHS have been conducted so far, which all have been included in the trend analysis, comprising the first wave (1990–1991), second wave (2006–2007), third wave (2012–2013), and fourth wave (2017–2018) of PDHS. Additionally, the Pakistan 2019 Maternal Mortality Survey (PMMS) has also been included [33–37]. Here, we will term PMMS as the fifth wave of PDHS for this research.

All these surveys have been carried out under a series of Demographic and Health Surveys, with financial funding from the US Agency for International Development and the technical support of the Pakistan Bureau of Statistics and ICF International. A series of PDHSs provides one of the largest datasets to academicians and researchers, entailing demographics and reproductive health–related variables, including ANC of married women of reproductive age (MWRAs) (15–49 years) across Pakistan [33–37].

Regarding the research design of PDHSs, two‐stage stratified random sampling techniques have been applied across all waves of PDHS to collect information from MWRAs. Firstly, the sampling units were selected based on geographical location (i.e., urban and rural). Secondly, the eligible households with MWRAs were selected for surveys [38]. Data was collected by trained field teams of interviewers, headed by supervisors and field editors, and closely monitored by a team of ICF and NIPS′ quality controllers and field coordinators. Simultaneously, double data entry, data editing, and data processing have been completed during the fieldwork [33–37].

During each wave of PDHSs, a set of questionnaires was used for data collection at the community, household, and individual levels (married women and married men). It is pertinent to mention that MWRAs were the key respondents within the first two waves, while married men were also interviewed to collect the couple data in the subsequent waves. Overall, the response rate of five waves of PDHS ranged between 93% and 96%. This study has used the women′s questionnaire dataset, entailing key questions from ANC [33–37].

Regarding sample size, there were 6910 of ever MWRAs during the first wave of PDHS, and 10,023 MWRAs during the second wave, followed by 13,558, 12,364, and 11,859 MWRAs during the third, fourth, and fifth waves of PDHS, were interviewed. Considering the research objectives, this study is limited to those MWRAs who had live births in the last 5 years preceding all five waves of PDHS. Thus, the sample size of women was limited to 4061, 5677, 7446, 6711, and 6472 MWRAs for the first, second, third, fourth, and fifth waves of PDHS, as illustrated in Table 1.

**Table 1:** Sample size of four waves of PDHS from 1990 to 2019.

	PDHS 1990–1991	PDHS 2006–2007	PDHS 2012–2013	PDHS 2017–2018	PMMS 2019
No. of ever‐married women of reproductive age interviewed	6910	10,023	13,558	12,364	11,859
No. of women who gave live birth in the previous 5 years	4061	5677	7446	6711	6472

### 3.2. Measures

#### 3.2.1. Outcome Variable

This research′s outcome variable was the women′s utilization of ANC service. For our research, the “adequate number of ANC visits” completed by MWRAs during their last pregnancy, according to the WHO standards [1], was taken as a dependent variable for all five waves of PDHS. It was inferred from the following question: “How many times did you receive antenatal care during this pregnancy?” The ANC visits were categorized into dichotomous responses: “adequate ANC (at least 4 or more ANC visits)” and “inadequate ANC (less than 4 or no ANC visits).” Though the WHO guidelines have been revised and emphasized to complete eight ANC visits, our research is consistent with previous research, considering the threshold of at least four ANC visits or more for analysis [24, 39].

#### 3.2.2. Explanatory Variables

Adopting the theory‐driven Andersen behavioral conceptual framework, we have identified the following factors as explanatory variables contributing to inequities in ANC utilization for our analysis. These variables were further grouped into the external environment, population characteristics (predisposing, enabling, and need factors), and health behavior, as detailed below.

##### 3.2.2.1. External Environment

These included provinces/regions (Punjab, Sindh, Khyber Pakhtunkhwa, Baluchistan, Islamabad, Federal Administrated Tribal Areas [FATA], and Gilgit Baltistan), type of geographical residence (urban/rural), and available means of transportation (own vehicle/no vehicle). The latter one was computed if a respondent had confirmed possession of any means of transportation, such as a bicycle, scooter/motorcycle, and car/truck [12].

##### 3.2.2.2. Population Characteristics

Firstly, these comprised of predisposing factors including respondent age in completed years (15–24, 25–34, and 35 years and above), current marital status (married, divorced, widowed, and separated), respondents′ and their husbands′ educational level (uneducated, primary, secondary, and higher), and respondents′ and their husbands′ occupation (professional/managerial/sales and services, agriculture, skilled, and unskilled workers).

Secondly, the enabling factors included household wealth index (poorest, poorer, middle, richer, and richest), respondents′ autonomy to seek healthcare (yes/no), visits to lady health workers (LHWs) in the last 12 months (yes/no), and exposure to mass media (yes/no). The latter variable was inferred based on the respondents′ frequency of listening to the radio, reading the newspaper, and watching television, which was computed into binary categories.

Thirdly, the need factors consisted of parity (1–2 children, 3–4 children, and 5 children or above), gravidity (0, 1–2, 3–4, and 5 or above), desire for more children (wants more children or no more children), and ever had a pregnancy loss (yes/no).

##### 3.2.2.3. Health Behavior

Lastly, the ANC service utilization–related health behavior comprised of providers of ANC, components of ANC, and use of contraception. The ANC providers were categorized into skilled providers (e.g., doctors and paramedics, e.g., nurse/midwife/LHV and medical assistant) and unskilled providers (e.g., traditional birth attendants/DAI, homeopaths, hakim, and LHWs) [40]. The components of ANC included monitoring of blood pressure, urine examination, blood sample, intake of supplements (iron tablets or syrups and drugs for intestinal parasites), and tetanus toxoid injections before pregnancy [21], which were coded into yes and no. The use of contraception was also coded into binary responses (yes and no).

It is pertinent to mention here that PMMS 2019 is the first national survey to measure maternal mortality in the country. It mainly collected data on births, deaths, and verbal autopsies, thus lacking data for some key variables as mentioned in the Results section [37].

### 3.3. Statistical Analysis

For the data analysis, women′s sample weights were applied, using the IBM SPSS Version 25. We carried out univariate descriptive analysis and presented frequency and percentage. Cross‐tabulation was performed for bivariate analysis to determine the relationship between outcome and explanatory variables, where a *p* value ≤ 0.05 was considered statistically significant. Given the cross‐sectional nature of the data, bivariate and multivariate logistic regressions were further applied to explore the association of adequate ANC visits with explanatory variables, where odds ratios (ORs) and adjusted odds ratios (AORs) at 95% confidence intervals (CIs) were calculated [19]. Moreover, the variance inflation factor was also calculated to assess multicollinearity among variables before regression, which was found to be higher with > 10; thus, a few variables were excluded for modeling [41].

Variables representing health service characteristics (providers of ANC, components of ANC, and current use of contraception) represent the quality and content of care received during ANC visits rather than factors influencing care‐seeking behavior. They were analyzed using descriptive statistics only to illustrate trends in service comprehensiveness over time.

## 4. Results

### 4.1. Descriptive Statistics of Respondents′ Key Characteristics

Table 2 illustrates the sociodemographic characteristics of ever MWRA aged 15–49 years, who gave birth during 5 years preceding the last five waves of PDHSs from 1991 to 2019. Here, Wave 1 represents PDHS 1990–1991, Wave 2 shows PDHS 2006–2007, Wave 3 denotes PDHS 2012–2013, Wave 4 indicates PDHS 2017–2018, and Wave 5 depicts PMMS 2019.

**Table 2:** Descriptive statistics of key characteristics of women of reproductive age 15–49 years, who gave birth during the 5 years preceding all waves of PDHSs (1990–2019).

Characteristics	PDHS 1990–1991		PDHS 2006–2007		PDHS 2012–2013		PDHS 2017–2018		PMMS 2019	
	*n* = 4061		*n* = 5677		*n* = 7446		*n* = 6711		*n* = 6472	
	*f*	%	*f*	%	*f*	%	*f*	%	*f*	%
Provinces										
Punjab	2441	60.1	3182	56.1	4180	56.1	3453	51.5	2617	40.4
Sindh	894	22.0	1404	24.7	1714	23.0	1571	23.4	1157	17.9
Khyber Pakhtunkhwa	567	14.0	827	14.6	1117	15.0	1101	16.4	1074	16.6
Baluchistan	159	3.9	264	4.6	348	4.7	377	5.6	305	4.7
Islamabad	—	—	—	—	31	0.4	54	0.8	—	—
FATA	—	—	—	—	—	—	156	2.3	—	—
Gilgit Baltistan	—	—	—	—	56	0.7	—	—	618	9.5
Azad Jammu Kashmir	—	—	—	—	—	—	—	—	701	10.8

Type of residence										
Urban	1184	29.1	1714	30.2	2244	30.1	2248	33.5	1882	29.1
Rural	2877	70.9	3962	69.8	5202	69.9	4468	66.5	4590	70.9

Women′s age										
15–24 years	983	24.2	1334	23.5	1748	23.5	1545	23.0	1760	27.2
25–34 years	2061	50.7	2952	52.0	4038	54.5	3725	55.5	3474	53.7
35 years and above	1017	25.0	1390	24.5	1659	22.3	1440	21.5	1238	19.1

Current marital status										
Married	4008	98.7	5596	98.6	7351	98.7	6604	98.4	6407	99.0
Widowed	33	0.8	37	0.7	39	0.5	63	0.9	32	0.5
Divorced	3	0.1	10	0.2	33	0.4	26	0.4	16	0.2
Separated	16	0.4	34	0.6	23	0.3	18	0.3	17	0.3

Women′s educational level										
No education	3214	79.2	3668	64.6	4155	55.8	3212	47.9	17	3.3
Primary	373	9.2	854	15.0	1230	16.5	1097	16.3	1099	31.3
Secondary	427	10.5	813	14.3	1380	18.5	1492	22.2	1409	40.1
Higher	47	1.2	341	6.0	682	9.2	911	13.6	888	25.3

Husband′s educational level^a^										
No education	1946	48.2	2007	35.5	2451	33.0	1889	28.7	—	—
Primary	698	17.3	935	16.5	1211	16.3	1085	16.5	—	—
Secondary	1213	30.0	1904	33.7	2547	34.3	2316	35.2	—	—
Higher	181	4.5	812	14.3	1216	16.4	1293	19.6	—	—
Women′s occupation^a^										

Not working/unemployed	3389	83.5	4026	71.0	5378	72.2	5528	82.4	—	—
Professional/clerical/sales & services	76	1.9	734	12.9	658	8.8	279	4.2	—	—
Agriculture	272	6.7	728	12.8	820	11.0	403	6.0	—	—
Manual or household worker	321	7.9	185	3.3	590	7.9	498	7.4	—	—

Husband′s occupation^a^										
Not working/unemployed	77	2.0	174	3.1	123	1.7	173	2.6	—	—
Professional/clerical/sales & services	1195	30.5	1999	35.2	2355	31.6	2119	32.2	—	—
Agriculture	1254	32.0	1185	20.9	1260	16.9	1154	17.5	—	—
Manual or household worker	1395	35.6	2316	40.8	3707	49.8	3142	47.7	—	—

Mass media exposure^a^										
Yes	760	24.0	—	—	3663	62.6	3855	61.1	2735	59.0
No	2404	76.0	—	—	2184	37.4	2454	38.9	1900	41.0

Available means of transportation										
No vehicle	2474	63.5	2523	53.7	3292	54.2	2256	42.3	2526	54.5
Own vehicle	1420	36.5	2178	46.3	2787	45.8	3076	57.7	2110	45.5

Household wealth index										
Poorest	—	—	1289	22.7	1698	22.8	1444	21.5	1079	23.3
Poorer	—	—	1194	21.0	1544	20.7	1299	19.4	1060	22.9
Middle	—	—	1099	19.4	1464	19.7	1371	20.4	1033	22.3
Richer	—	—	1066	18.8	1469	19.7	1349	20.1	845	18.2
Richest	—	—	1029	18.1	1272	17.1	1248	18.6	618	13.3

Visits of LHW in the last 12 months^a^										
Yes	—	—	1533	27.0	2975	56.8	4147	61.8	1897	29.3
No	—	—	4143	73.0	2260	43.2	2564	38.2	4575	70.7

Autonomy to seek healthcare^a^										
Yes	—	—	—	—	2982	40.1	3024	45.1	—	—
No	—	—	—	—	4451	59.9	3683	54.9	—	—

Parity										
1–2 children	1245	30.7	2000	35.2	2885	38.7	2749	41.0	2670	41.3
3–4 children	1142	28.1	1648	29.0	2249	30.2	2183	32.5	2140	33.1
5 children or above	1674	41.2	2029	35.7	2312	31.1	1780	26.5	1662	25.7

Gravidity^a^										
0	71	1.7	100	1.8	80	1.1	83	1.2	—	—
1–2	1360	33.5	2138	37.7	3149	42.3	2944	43.9	—	—
3–4	1294	31.9	1737	30.6	2306	31.0	2222	33.1	—	—
5 or above	1336	32.9	1702	30.0	1912	25.7	1463	21.8	—	—

Desire for more children										
Wants more children	1748	43.7	2647	46.7	3592	49.0	3115	47.2	141	75.9
Wants no more children	2254	56.3	3023	53.3	3745	51.0	3480	52.8	45	24.1

Ever had a pregnancy loss^a^										
Yes	—	—	1352	23.8	2512	33.7	2166	32.3	1954	30.2
No	—	—	4320	76.2	4935	66.3	4545	67.7	4517	69.8

Current use of contraceptive methods										
No method	3586	88.3	4007	70.6	4672	62.7	4290	63.9	3413	52.7
Traditional method	131	3.2	489	8.6	782	10.5	630	9.4	571	8.8
Modern method	344	8.5	1181	20.8	1992	26.7	1791	26.7	2488	38.4

*Note:* Superscript “a” indicates nonavailability of data.

Results informed that the majority of the ever MWRAs were aged 25–34 years (50.7%, 52.0%, 54.5%, 55.5%, and 53.7%) in all the waves of PDHS. A significant proportion of the women were found to be married (98.7%, 98.6%, 98.7%, 98.4%, and 99%), belonging to the provinces of Punjab (60.1%, 56.1%, 56.1%, 51.5%, and 40.4%) in all the waves of PDHS.

In terms of education, a larger number of women had no formal education (79.2%, 64.6%, 55.8%, and 47.9%) across the first four waves of PDHSs in contrast to the fifth wave (3.3%), while a higher proportion of husbands had attained a secondary level of education (30.0%, 33.7%, 34.3%, and 35.2%) during first four waves of PDHS.

Further results revealed that the majority of the women belonged to the not working/unemployed occupation (83.5%, 71.0%, 72.2%, and 82.4%), whereas a higher number of their husbands also worked in manual or household work (35.6%, 40.8%, 49.8%, and 47.7%) during the first four waves of PDHS.

Regarding respondents′ exposure to mass media, more than half of the respondents (62.6%, 61.1%, and 59%) had access to any means of mass media (i.e., radio, TV, or newspaper) in the last three waves of PDHS. Further, a significant proportion of women declined the ownership of any vehicle for transportation (e.g., bicycle, scooter/motorcycle, and car/truck) (63.5%, 53.7%, 54.2%, 42.3%, and 54.5%) from the first to the fifth waves of PDHS. The results highlighted that more women belonged to the poorest household quintiles (22.7%, 22.8%, 21.5%, and 23.3%) during the last four waves of PDHSs.

According to the findings, a large proportion of women (59.9% and 54.9%) reported that they had no autonomy to seek healthcare, while more than half of the respondents (56.8% and 61.8%) confirmed that LHWs visited them during the last 12 months in Waves 3 and 4, respectively. Furthermore, results indicated that the majority of women had five children or above (41.2%, 35.7%, and 31.1%) during the first three waves of PDHS, similar to their gravidity with one to two children (33.5%, 37.7%, 42.3%, and 43.9%), particularly during the first four waves. Furthermore, results indicated that a significant percentage of women did not desire more children (56.3%, 53.3%, 51.0%, and 52.8%) across the first four waves of PDHSs. It is also pertinent to mention that most of the women had not gone through pregnancy loss (76.2%, 66.3%, 67.7%, and 69.8%) during the last four waves of PDHSs. Lastly, a larger number of women were not using any contraceptive method presently (88.3%, 70.6%, 62.7%, 63.9%, and 52.7%) in all the waves of PDHSs.

### 4.2. ANC‐Related Characteristics

**Figure 2:**
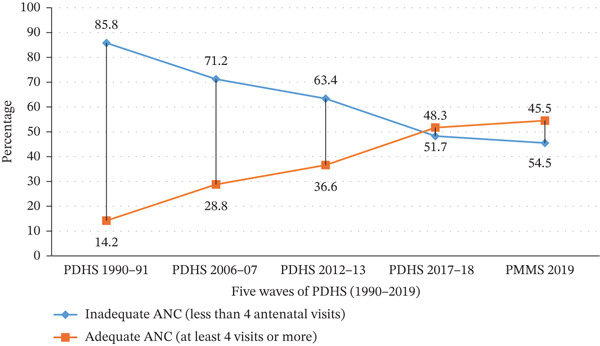
Trend line of the number of ANC visits by respondents from 1990 to 2019.

The findings of Table 3 highlighted the respondents′ ANC‐related characteristics over the period from 1990 to 2019. Figure 2 shows an upward trend and a gradual increase in the utilization of an adequate number of ANC visits from 14.2% to 54.5% of Pakistani women during the last three decades. Consequently, a steady decrease is evident for an inadequate number of ANC visits (85.8%, 71.2%, 63.4%, 51.7%, and 45.5%). On average, the mean value of ANC visits increased from 2.7 visits to 4.6 visits from 1990 to 2019, which is encouraging.

**Table 3:** ANC‐related characteristics of women of reproductive age 15–49 years, who gave birth during the 5 years preceding all waves of PDHSs (1990–2019).

Characteristics	PDHS 1990–1991		PDHS 2006–2007		PDHS 2012–2013		PDHS 2017–2018		PMMS 2019	
	*n* = 4061		*n* = 5677		*n* = 7446		*n* = 6711		*n* = 6472	
	*f*	%	*f*	%	*f*	%	*f*	%	*f*	%
Adequate number of ANC visits										
Inadequate ANC (less than 4 antenatal visits)	3410	85.8	3987	71.2	4713	63.4	3231	48.3	2681	45.5
Adequate ANC (at least 4 visits or more)	564	14.2	1611	28.8	2723	36.6	3452	51.7	3213	54.5

Provider of ANC										
Skilled providers	1212	98.5	3398	61.2	5205	72.4	5576	85.9	5001	98.4
Unskilled providers	18	1.5	289	5.1	273	3.7	198	2.9	81	1.6

Component of ANC^a^										
Monitoring blood pressure	—	—	2925	79.7	4838	86.0	5268	89.4	5293	89.7
Urine sample	—	—	1809	49.3	3425	60.8	4172	70.8	4072	69.0
Blood sample	—	—	1607	43.8	3134	55.7	4150	70.4	4327	73.3
Supplements (iron tablets/syrup)	—	—	2457	43.7	3329	44.9	3924	58.7	4364	67.6
Drugs for intestinal parasites	—	—	—	—	186	2.5	122	1.8	93	1.4
Tetanus toxoid (TT) injections	—	—	554	21.8	633	21.0	582	24.7	868	35.9

*Note:* Superscript “a” indicates nonavailability of data.

Among the women who availed the ANC services, the majority had consulted skilled healthcare providers during ANC (98.5%, 61.2%, 72.4%, 85.9%, and 98.4%) from the first to the fifth waves of PDHSs. Regarding various components of ANC, in most cases from the second to the fifth waves, women′s blood pressure was monitored, and their urine and blood samples were examined. Furthermore, they were also provided with supplements along with tetanus toxoid injections. However, very few respondents were given drugs for intestinal parasites in the last three waves of PDHS.

### 4.3. Association Between Adequate ANC Visits and Key Characteristics

Table 4 presents the association between adequate numbers of ANC visits and key characteristics of women of reproductive age 15–49 years, who gave birth during the last 5 years preceding PDHSs (1990–2019). The table shows both the percentages of adequate ANC within each characteristic category and the strength of association (ORs with 95% CIs) from bivariate logistic regression analysis.

**Table 4:** Association between adequate ANC visits and characteristics of women of reproductive age 15–49 years, who gave birth during the 5 years preceding DHSs (1990–2019).

Characteristics	PDHS 1990–1991				PDHS 2006–2007				PDHS 2012–2013				PDHS 2017–2018				PMMS 2019			
	*n* = 4061				*n* = 5677				*n* = 7446				*n* = 6711				*n* = 6472			
	%	OR	CI (95%)	*p* value	%	OR	CI (95%)	*p* value	%	OR	CI (95%)	*p* value	%	OR	CI (95%)	*p* value	%	OR	CI (95%)	*p* value
Provinces																				
Punjab	12.0	1			29.7	1			38.5	1			56.5	1			57.6	1		
Sindh	26.3	2.63 ^∗^	2.16–3.19	**< 0.01**	36.8	1.37 ^∗^	1.20–1.57	**< 0.01**	44.5	1.28 ^∗^	1.14–1.43	**< 0.01**	54.1	0.91	0.80–1.02	0.12	59.7	1.08	0.93–1.26	0.26
Khyber Pakhtunkhwa	6.7	0.52 ^∗^	0.37–0.75	**< 0.01**	18.2	0.52 ^∗^	0.43–0.63	**< 0.01**	24	0.50 ^∗^	0.43–0.58	**< 0.01**	45	0.63 ^∗^	0.54–0.72	**< 0.01**	47.6	0.66 ^∗^	0.57–0.77	**< 0.01**
Baluchistan	6.6	0.54	0.27–1.06	0.07	7.7	0.20 ^∗^	0.12–0.32	**< 0.01**	12.2	0.22 ^∗^	0.16–0.31	**< 0.01**	23.1	0.23 ^∗^	0.18–0.29	**< 0.01**	39.7	0.48 ^∗^	0.36–0.63	**< 0.01**
Gilgit Baltistan	—	—	—	—	—	—	—	—	30.9	0.71	0.40–1.26	0.25	—	—	—	—	45.3	0.60 ^∗^	0.50–0.73	**< 0.01**
Islamabad	—	—	—	—	—	—	—	—	81.3	7.31 ^∗^	2.92–18.26	**< 0.01**	79.6	3.17 ^∗^	1.61–6.24	**< 0.01**	—	—	—	**—**
FATA	—	—	—	—	—	—	—	—	—	—	—	—	25.6	0.26 ^∗^	0.18–0.38	**< 0.01**	—	—	—	**—**
Azad Jammu Kashmir	—	—	—	—	—	—	—	—	—	—	—	—		—	—	**—**	56.6	0.95	0.80–1.13	0.6
Type of residence																				
Rural	36.0	1			49	1			61.7	1			71.2	1			71.3	1		
Urban	5.2	0.09 ^∗^	0.07–0.11	**< 0.01**	20	0.26 ^∗^	0.23–0.29	**< 0.01**	25.8	0.21 ^∗^	0.19–0.23	**< 0.01**	41.8	0.29 ^∗^	0.26–0.32	**< 0.01**	47.2	0.35 ^∗^	0.31–0.40	**< 0.01**

Women′s age																				
15–24 years	14.5	1			27.7	1			37	1			49.2	1			53.1	1		
25–34 years	16.3	1.14	0.92–1.42	0.21	32.1	1.23 ^∗^	1.07–1.42	**< 0.01**	39.4	1.1	0.98–1.24	0.08	55	1.26 ^∗^	1.11–1.42	**< 0.01**	56.3	1.13 ^∗^	1.01–1.28	**0.03**
35 years and above	9.7	0.62 ^∗^	0.47–0.82	**< 0.01**	22.7	0.76 ^∗^	0.64–0.91	**< 0.01**	29.4	0.70 ^∗^	0.61–0.81	**< 0.01**	45.7	0.87 ^∗^	0.75–1.00	**0.05**	51.4	0.93	0.80–1.08	0.38

Current marital status																				
Widowed	14.2	1			29	1			36.7	1			51.8	1			54.6	1		
Separated	6.1	0.45	0.11–1.72	0.24	16.7	0.43	0.16–1.11	0.08	25.6	0.21 ^∗^	0.06–0.79	**0.02**	32.8	0.45 ^∗^	0.26–0.77	**< 0.01**	34.4	0.45 ^∗^	0.22–0.94	**0.03**
Divorced	75	2.24	0.73–6.81	0.15	27.3	0.49	0.20–1.19	0.11	39.4	0.6	0.29–1.23	0.16	42.3	0.67	0.31–1.47	0.32	33.3	0.41	0.14–1.20	0.1
Married	25	19.23 ^∗^	1.53–241.1	**< 0.01**	14.7	0.83	0.20–3.43	0.8	13	1.15	0.57–2.33	0.68	64.7	1.71	0.64–4.58	0.28	70.6	1.78	0.64–4.95	0.26

Women′s educational level																				
No education	7.3	1			17.6	1			20.1	1			31.8	1			49.1	1		
Primary	20.4	3.30 ^∗^	2.47–4.40	**< 0.01**	32.5	2.24 ^∗^	1.90–2.66	**< 0.01**	39.1	2.54 ^∗^	2.22–2.92	**< 0.01**	53.3	2.45 ^∗^	2.12–2.81	**< 0.01**	55.9	1.29	0.87–1.91	0.19
Secondary	52.8	14.37 ^∗^	11.37–18.16	**< 0.01**	55.5	5.83 ^∗^	4.95–6.87	**< 0.01**	61.2	6.25 ^∗^	5.47–7.13	**< 0.01**	71.6	5.34 ^∗^	4.74–6.21	**< 0.01**	67	2.08 ^∗^	1.41–3.06	**< 0.01**
Higher	84.8	67.76 ^∗^	30.32–151.4	**< 0.01**	76.2	14.88 ^∗^	11.42–19.40	**< 0.01**	83	19.37 ^∗^	15.64–23.98	**< 0.01**	87	14.33 ^∗^	11.65–17.63	**< 0.01**	81	4.48 ^∗^	2.98–6.73	**< 0.01**

Husband′s educational level																				
No education	6.3	1			15.9	1			21.1	1			30.7	1			**—**	1		
Primary	8.7	1.42 ^∗^	1.03–1.96	**0.03**	21.2	1.42 ^∗^	1.16–1.73	**< 0.01**	27.9	1.43 ^∗^	1.22–1.68	**< 0.01**	43.5	1.73 ^∗^	1.48–2.02	**< 0.01**	**—**	**—**	**—**	**—**
Secondary	25	4.95 ^∗^	3.94–6.21	**< 0.01**	35.6	2.91 ^∗^	2.50–3.39	**< 0.01**	44.1	2.93 ^∗^	2.59–3.32	**< 0.01**	60	3.38 ^∗^	2.97–3.84	**< 0.01**	**—**	**—**	**—**	**—**
Higher	46.6	12.98 ^∗^	9.16–18.37	**< 0.01**	53.7	6.12 ^∗^	5.09–7.35	**< 0.01**	61.1	5.86 ^∗^	5.04–6.82	**< 0.01**	75.3	6.87 ^∗^	5.85–8.06	**< 0.01**	**—**	**—**	**—**	**—**

Women′s occupation																				
Not working/unemployed	14.5	1			31.6	1			40.1	1			53.8	1			—	1		
Professional/clerical/sales & services	26.8	2.19 ^∗^	1.28–3.73	**< 0.01**	29.6	0.91	0.76–1.08	0.28	48.3	1.40 ^∗^	1.19–1.64	**< 0.01**	66.1	1.66 ^∗^	1.29–2.15	**< 0.01**	—	—	—	—
Agriculture	5	0.30 ^∗^	0.17–0.54	**< 0.01**	13.2	0.33 ^∗^	0.26–0.41	**< 0.01**	16	0.28 ^∗^	0.23–0.34	**< 0.01**	22.3	0.24 ^∗^	0.19–0.31	**< 0.01**	—	—	—	—
Manual or household worker	15.2	1.04	0.75–1.44	0.79	25.4	0.72	0.51–1.02	0.06	20.8	0.39 ^∗^	0.32–0.48	**< 0.01**	44.1	0.67 ^∗^	0.56–0.81	**< 0.01**	—	—	—	—

Husband′s occupation																				
Not working/unemployed	11.7	1			27.2	1			25.2	1			47.1	1			—	1		
Professional/clerical/sales & services	23.5	2.19 ^∗^	1.09–4.40	**0.02**	38.2	1.66 ^∗^	1.17–2.36	**< 0.01**	48.9	2.83 ^∗^	1.87–4.28	**< 0.01**	65.1	2.11 ^∗^	1.54–2.89	**< 0.01**	—	—	—	—
Agriculture	4.8	0.35 ^∗^	0.17–0.74	**< 0.01**	17.2	0.56 ^∗^	0.38–0.81	**< 0.01**	21.2	0.79	0.52–1.22	0.3	32.8	0.55 ^∗^	0.40–0.76	**< 0.01**	—	—	—	—
Manual or household worker	14.4	1.21	0.60–2.43	0.58	26.8	0.98	0.69–1.40	0.95	34.4	1.55 ^∗^	1.02–2.34	**0.03**	50	1.13	0.83–1.54	0.42	**—**	**—**	**—**	**—**

Mass media exposure																				
No	4.4	1			—	—	—	—	15.7	1			32.9	1			41.1	1		
Yes	14.4	3.62 ^∗^	2.72–4.80	**< 0.01**	—	—	—	—	38	3.30 ^∗^	2.88–3.77	**< 0.01**	61.4	3.24 ^∗^	2.91–3.61	**< 0.01**	59.9	0.46 ^∗^	0.41–0.53	**< 0.01**

Available means of transportation																				
No vehicle	11.5	1			76.4	1			27.1	1			42.4	1			47.6	1		
Own vehicle	16.5	1.52 ^∗^	1.26–1.84	**< 0.01**	70.9	1.32 ^∗^	1.16–1.51	**< 0.01**	41.6	1.91 ^∗^	1.72–2.13	**< 0.01**	54.1	1.60 ^∗^	1.43–1.79	**< 0.01**	58.6	1.55 ^∗^	1.37–1.76	**< 0.01**

Household wealth index																				
Poorest	—	—	—	—	10.3	1			12.9	1			22.8	1			30.2	1		
Poorer	—	—	—	—	14.5	1.48 ^∗^	1.16–1.88	**< 0.01**	21.7	1.86 ^∗^	1.55–2.25	**< 0.01**	34.3	1.76 ^∗^	1.49–2.08	**< 0.01**	42	1.68 ^∗^	1.38–2.04	**< 0.01**
Middle	—	—	—	—	22.3	2.49 ^∗^	1.98–3.14	**< 0.01**	31	3.02 ^∗^	2.52–3.62	**< 0.01**	50.1	3.39 ^∗^	2.88–3.99	**< 0.01**	51.5	2.46 ^∗^	2.03–2.98	**< 0.01**
Richer	—	—	—	—	39	5.57 ^∗^	4.47–6.94	**< 0.01**	49.9	6.70 ^∗^	5.62–7.98	**< 0.01**	69	7.54 ^∗^	6.37–8.93	**< 0.01**	67.5	4.82 ^∗^	3.91–5.93	**< 0.01**
Richest	—	—	—	—	64.8	16.03 ^∗^	12.83–20.01	**< 0.01**	77.6	23.33 ^∗^	19.22–28.32	**< 0.01**	86.4	21.50 ^∗^	17.54–26.37	**< 0.01**	82.6	11.0 ^∗^	8.53–14.2	**< 0.01**
Visits of LHW in the last 12 months																				

No	—	—	—		28.6	1			39.4	1			47.1	1			51.5	**1**		
Yes	—	—	—		29.2	1.02	0.90–1.17	0.67	35.7	0.85 ^∗^	0.76–0.95	**< 0.01**	54.5	1.34 ^∗^	1.21–1.48	**< 0.01**	61.3	0.67 ^∗^	0.59–0.75	**< 0.01**

Autonomy to seek healthcare																				
No	—	—	—			—	—		43.7	1			60.7	1			—	1		
Yes	—	—	—			—	—		26	0.45 ^∗^	0.41–0.50	**< 0.01**	40.7	0.44 ^∗^	0.40–0.49	**< 0.01**	—	—	—	—

Parity																				
5 children or above	9.7	1			21.4	1			23.8	1			37.2	1			39.7	**1**		
3–4 children	16.9	0.94	0.76–1.16	0.58	30	0.78 ^∗^	0.68–0.90	**< 0.01**	36.2	0.63 ^∗^	0.56–0.71	**< 0.01**	50.6	0.62 ^∗^	0.56–0.70	**< 0.01**	55.4	0.78 ^∗^	0.66–0.84	**< 0.01**
1–2 children	17.8	0.49 ^∗^	0.39–0.61	**< 0.01**	35.2	0.50 ^∗^	0.43–0.57	**< 0.01**	47.2	0.34 ^∗^	0.30–0.39	**< 0.01**	61.9	0.36 ^∗^	0.32–0.41	**< 0.01**	62.4	0.39 ^∗^	0.34–0.45	**< 0.01**

Gravidity																				
5 or above	10.1	1			20.4	1			22.8	1			36.9	1			—	**1**		
3–4	14.4	4.80 ^∗^	1.36–16.95	**0.01**	29.7	1.14	0.91–2.30	0.11	34.2	1.95 ^∗^	1.21–3.15	**< 0.01**	49.6	2.15 ^∗^	1.38–3.35	**< 0.01**	—	—	—	**—**
1–2	18.4	3.48 ^∗^	1.01–12.65	**0.04**	34.7	1.15	0.72–1.83	0.55	46.9	1.15	0.71–1.86	0.56	60.8	1.36	0.87–2.12	0.17	—	—	—	—
0	5.3	2.38	0.67–8.47	0.17	27.1	0.7	0.43–1.12	0.13	31.3	0.65	0.40–1.06	0.08	42.2	0.81	0.51–1.27	0.36	—	—	—	—

Desire for more children																				
Wants no more children	13.9	1			28.7	1			33.8	1			48.9	1			42.9	**1**		
Wants more children	14.4	0.96	0.80–1.15	0.69	29	0.98	0.87–1.10	0.83	39.8	0.77 ^∗^	0.70–0.84	**< 0.01**	55	0.78 ^∗^	0.71–0.86	**< 0.01**	63.2	0.44 ^∗^	0.22–0.89	**0.02**

Ever had a pregnancy loss																				
No	—	—			27.1	1			35.9	1			51.1	1			55.4	**1**		
Yes	—	—	—	—	33.9	1.38 ^∗^	1.21–1.57	**< 0.01**	38	1.09	0.99–1.20	0.07	52.9	1.07	0.97–1.19	0.15	52.3	1.13 ^∗^	1.01–1.26	**0.02**

*Note:* % = percentages represent the proportion of women within each characteristic category who achieved adequate ANC visits (≥ 4 visits); — = data not available in specific survey waves; 1 = OR reference categories. Bold values indicate statistically significant associations (*p* < 0.05).

Abbreviations: CI, confidence interval; OR, odds ratio (from bivariate logistic regression).

^∗^(*p* ≤ 0.05) = *p* values from bivariate logistic regression.

Results showed statistically significant associations (*p* ≤ 0.05) between adequate numbers of ANC visits and multiple factors across all survey waves, including provinces/regions, type of residence, women′s age, both women′s and husband′s education and occupation, mass media exposure, available means of transportation, household wealth index, autonomy to seek healthcare, parity, and gravidity.

Strong statistical associations were also observed with current marital status during the first, third, fourth, and fifth waves of PDHS. Furthermore, significant relationships were found with household wealth index in the last four waves, with LHWs′ visits and desire for more children in the last three waves, and with pregnancy loss in Waves 2 and 5 of PDHS.

More specifically, the odds of adequate ANC visits were significantly higher among (OR: 19.23, 0.83, 1.15, 1.71, and 1.78 across the five waves, respectively) women in the younger age group of 25–34 years (OR: 1.14, 1.23, 1.10, 1.26, and 1.13), those with higher educational attainment (OR: 67.76, 14.88, 19.37, 14.33, and 4.88), and women with parity of three to four children (OR: 0.94, 0.78, 0.63, 0.62, and 0.78) across all five waves. The probability of availing adequate ANC was substantially higher among households in the richest wealth quintile (OR: 16.03, 23.33, 21.50, and 11.0) in the last four waves. Women with exposure to mass media and gravidity of one to two children were more likely to achieve adequate ANC visits during Waves 1, 3, and 4 of PDHS.

### 4.4. Multivariate Logistic Regression Analysis

Table 5 exhibits multivariate logistic regression of an adequate number of ANC visits with key characteristics of women of reproductive age 15–49 years, who gave birth during the last 5 years preceding PDHSs (1990–2019).

**Table 5:** Multivariate logistic regression of adequate numbers of ANC visits with characteristics of women of reproductive age 15–49 years, who gave birth during the 5 years preceding PDHSs (1990–2019).

Characteristics	PDHS 1990–1991			PDHS 2006–2007			PDHS 2012–2013			PDHS 2017–2018			PMMS 2019		
	*n* = 4061			*n* = 5677			*n* = 7446			*n* = 6711			*n* = 6472		
	AOR	CI (95%)	*p*value	AOR	CI (95%)	*p*value	AOR	CI (95%)	*p*value	AOR	CI (95%)	*p*value	AOR	CI (95%)	*p*value
Provinces															
Punjab	1			1			1			1			1		
Sindh	2.71 ^∗^	1.89–3.89	**< 0.01**	1.85 ^∗^	1.53–2.23	**< 0.01**	1.63 ^∗^	1.29–2.05	**< 0.01**	1.59 ^∗^	1.33–1.89	**< 0.01**	0.17	0.01–2.18	0.17
Khyber Pakhtunkhwa	0.75	0.44–1.29	0.30	0.66 ^∗^	0.51–0.84	**< 0.01**	0.84	0.64–1.10	0.21	1.05	0.86–1.28	0.60	0.38	0.02–5.75	0.49
Baluchistan	1.69	0.73–3.88	0.21	0.46 ^∗^	0.26–0.80	**< 0.01**	0.88	0.45–1.72	0.71	0.52 ^∗^	0.36–0.76	**< 0.01**	0.13	0.00–9.13	0.34
Gilgit Baltistan	—	—		—	—		2.53 ^∗^	1.12–5.71	**0.02**	—	—		0.11	0.00–2.93	0.19
Islamabad	—	—		—	—		3.57	0.57–22.3	0.17	1.60	0.66–3.90	0.29	—	—	—
FATA	—	—		—	—		—	—		1.03	0.67–1.58	0.88	—	—	—
Azad Jammu Kashmir	—	—		—	—		—	—		—	—		0.17	0.00–5.97	0.33
Type of residence															
Rural	1			1			1			1			1		
Urban	0.26 ^∗^	0.18–0.36	**< 0.01**	0.85	0.70–1.03	0.10	0.65 ^∗^	0.52–0.82	**< 0.01**	0.89	0.75–1.06	0.19	1.18	0.19–7.30	0.85
Woman′s age															
15–24 years	1			1			1			1			1		
25–34 years	1.16	0.76–1.75	0.48	1.15	0.93–1.42	0.19	1.31 ^∗^	1.03–1.66	**0.02**	1.25 ^∗^	1.05–1.50	**0.01**	—	—	—
35 years and above	1.01	0.57–1.82	0.94	1.10	0.86–1.46	0.48	1.26	0.91–1.74	0.15	1.10	0.87–1.40	0.41	—	—	—
Women′s educational level															
No education	1			1			1			1			1		
Primary	—	—	—	1.24 ^∗^	0.99–1.54	**0.05**	1.34 ^∗^	1.05–1.69	**0.01**	1.58 ^∗^	1.32–1.90	**< 0.01**	—	—	—
Secondary	—	—	**—**	1.96 ^∗^	1.54–2.49	**< 0.01**	2.03 ^∗^	1.56–2.63	**< 0.01**	1.96 ^∗^	1.61–2.39	**< 0.01**	—	—	—
Higher	—	—	—	2.96 ^∗^	1.99–4.39	**< 0.01**	3.46 ^∗^	2.18–5.48	**< 0.01**	3.06 ^∗^	2.22–4.23	**< 0.01**	—	—	—
Husband′s educational level															
No education	1			1			1			1			1		
Primary	1.01	0.64–1.59	0.94	0.97	0.77–1.23	0.84	0.92	0.71–1.18	0.52	1.14	0.94–1.38	0.09	—	—	—
Secondary	2.55 ^∗^	1.77–3.66	**< 0.01**	1.36 ^∗^	1.11–1.66	**< 0.01**	1.21	0.96–1.51	0.09	1.46 ^∗^	1.23–1.73	**0.03**	**—**	**—**	**—**
Higher	2.79 ^∗^	1.22–6.37	**0.01**	1.67 ^∗^	1.26–2.20	**< 0.01**	1.19	0.86–1.65	0.27	1.60 ^∗^	1.27–2.02	**< 0.01**	—	—	—
Mass media exposure															
No	1			—	—		1			1			1		
Yes	1.98 ^∗^	1.42–2.74	**< 0.01**	—	—		1.31 ^∗^	1.07–1.61	**< 0.01**	1.24 ^∗^	1.07–1.45	**< 0.01**	0.44	0.07–2.63	0.37
Available means of transportation															
No vehicle	1			1			1			1			1		
Own vehicle	0.96	0.68–1.35	0.84	0.83 ^∗^	0.71–0.98	**0.03**	1.07	0.89–1.28	0.45	0.87 ^∗^	0.75–1.00	**0.05**	5.22	0.70–38.8	0.10
Household wealth index															
Poorest	—	—		1			1			1			1		
Poorer	—	—		1.53 ^∗^	1.17–2.00	**< 0.01**	1.72 ^∗^	1.31–2.25	**< 0.01**	1.71 ^∗^	1.40–2.09	**< 0.01**	—	—	—
Middle	—	—		2.41 ^∗^	1.84–3.18	**< 0.01**	1.94 ^∗^	1.44–2.61	**< 0.01**	2.40 ^∗^	1.92–3.00	**< 0.01**	—	—	—
Richer	—	—		3.73 ^∗^	2.78–5.00	**< 0.01**	2.80 ^∗^	2.00–3.91	**< 0.01**	3.77 ^∗^	2.87–4.94	**< 0.01**	—	—	—
Richest	—	—		8.29 ^∗^	5.83–11.78	**< 0.01**	5.59 ^∗^	3.55–8.81	**< 0.01**	7.41 ^∗^	5.16–10.06	**0.05**	—	—	—
Visits of LHW in the last 12 months															
No	—	—		1			1			1			1		
Yes	—	—		0.90	0.76–1.07	0.24	0.86	0.73–1.03	0.11	1.17 ^∗^	1.01–1.35	**0.02**	—	—	—
Autonomy to seek healthcare															
No	—	—		—	—		1			1			1		
Yes	—	—		—	—		—	—	**—**	—	—	—	—	—	—
Parity															
5 children or above	1			1			1			1			1		
3–4 children	2.43 ^∗^	1.31–4.51	**< 0.01**	0.82	0.55–1.22	0.33	1.00	0.67–1.48	0.99	0.66 ^∗^	0.48–0.92	**0.01**	0.42	0.05–3.51	0.43
1–2 children	1.36	0.54–3.37	0.50	1.12	0.68–1.85	0.64	1.57	0.92–2.69	0.09	0.73	0.47–1.11	0.14	0.36	0.04–2.83	0.33
Gravidity															
5 or above	1			1			1			1			1		
1–2	2.80	0.48–16.25	0.24	1.42	0.72–2.80	0.31	1.45	0.57–3.70	0.42	1.39	0.78–2.48	0.25	—	—	—
3–4	0.93	0.15–5.81	0.94	1.06	0.48–2.34	0.88	0.69	0.25–1.93	0.48	1.46	0.75–2.84	0.26	—	—	—
0	1.25	0.17–8.94	0.81	0.70	0.30–1.67	0.43	0.34 ^∗^	0.11–1.01	**0.05**	1.24	0.60–2.57	0.54	—	—	—
Desire for more children															
Wants no more children	1			1			1			1			1		
Wants more children	0.88	0.62–1.27	0.52	1.35 ^∗^	1.11–1.64	**< 0.01**	1.20	0.96–1.50	0.09	0.97	0.83–1.13	0.73	0.32	0.04–2.33	0.26
Ever had a pregnancy loss															
No				1			1			1			1		
Yes	—	—	—	1.50 ^∗^	1.26–1.78	**< 0.01**	1.42 ^∗^	1.19–1.70	**< 0.01**	1.16 ^∗^	1.01–1.34	**0.03**	0.79	0.15–4.17	0.78

*Note:* 1 = OR reference categories; — = data not available in specific survey waves. Bold values indicate statistically significant associations (*p* < 0.05).

Abbreviations: AOR, adjusted odds ratio; CI, confidence interval.

^∗^(*p* ≤ 0.05) = *p* values.

Findings revealed a variation between the significance of an adequate number of ANC visits, with multiple factors contributing to inequities. Results informed a significant association of an adequate number of ANC visits with women′s higher level of education (AOR = 2.96, 95% CI: 1.99–4.39; AOR = 3.46, 95% CI: 2.18–5.48; AOR = 3.06, 95% CI: 2.22–4.23) in Waves 2, 3, and 4 and husband′s higher level of education (AOR = 2.79, 95% CI: 1.22–6.37; AOR = 1.67, 95% CI: 1.26–2.20; AOR = 1.60, 95% CI: 1.27–2.02) during Waves 1, 2, and 4 of PDHS. Further, the adequacy of ANC visits was seen statistically significant with the richest wealth quintile (AOR = 1.75, 95% CI: 0.81–3.76; AOR = 2.74, 95% CI: 0.92–8.40; AOR = 2.23, 95% CI: 0.97–5.10) in Waves 2, 3, and 4 and a gravidity of one to two children (AOR = 1.60, 95% CI: 0.25–10.05; AOR = 0.99, 95% CI: 0.24–4.09; AOR = 1.61, 95% CI: 0.31–8.30) in the last three waves of PDHSs.

Further, a higher likelihood of an adequate number of ANC visits was found among women of 25–34 years of age (AOR = 2.31, 95% CI: 1.03–1.66; AOR = 1.25, 95% CI: 1.05–1.50) in Waves 3 and 4 of PDHS.

Furthermore, higher AOR was seen for the women residing in Sindh Province (AOR = 2.71, 95% CI: 1.89–3.89; AOR = 1.85, 95% CI: 1.53–2.23; AOR = 1.63, 95% CI: 1.29–2.05; AOR = 1.59, 95% CI: 1.33–1.89) in Waves 1, 2, 3, and 4; belonging to the richest wealth quintile (AOR = 8.29, 95% CI: 5.83–11.78; AOR = 5.59, 95% CI: 3.55–8.81; AOR = 7.41, 95% CI: 5.16–10.06) in Waves 2, 3, and 4; and having a parity of three to four children (AOR = 2.43, 95% CI: 1.31–4.51; AOR = 0.66, 95% CI: 0.48–0.92) in Waves 1 and 4, respectively.

## 5. Discussion

The subject of ANC utilization is of utmost importance to understand how often women in Pakistan utilize prenatal services to avert pregnancy complications and acquire health education for achieving optimal maternal and newborn health. The present study is aimed at analyzing the trends and inequities related to ANC utilization within three dimensions (i.e., external environmental, population characteristics, and health behaviors) among women of reproductive age 15–49 years in Pakistan across the last five waves of PDHS from 1990 to 2019.

The research witnessed an upward trend in the utilization of adequate ANC visits, with an increase from 14.2% to 54.5% from 1990 to 2019 in the country, highlighting the positive contribution of the government, health facilities, and healthcare providers. Similar trends have been observed at the regional/provincial level, particularly in the case of Islamabad, Punjab, Sindh, and Khyber Pakhtunkhwa; however, the lower utilization of adequate ANC visits was seen for Baluchistan and FATA. The lower utilization of maternal services within some geographical areas may be attributed to health inequalities [42], particularly in terms of a lack of infrastructure and skilled providers, and limited health education among women [18, 43], which requires immediate attention. Further, an improvement in essential components of ANC, including medical check‐ups and laboratory tests, was also found, highlighting the comprehensiveness of optimal ANC service utilization. It also corresponds to the research conducted on maternal health service utilization in Pakistan [44].

Findings informed that adequate numbers of ANC utilization had a strong association with population characteristics of MWRAs aged 15–49 years. Particularly, it was found to be higher among 25–34‐year‐old women who attained higher education and were employed in professional/managerial occupations. These findings related to women′s age, education, and occupation are consistent with the studies conducted on maternal health utilization in sub‐Saharan Africa [45], Nigeria [46], Madagascar [7], Ethiopia [23], and Pakistan [13, 38, 47]. A variation of inequities related to ANC utilization was also manifested from other key factors, such as poor wealth quintile, nonavailability of transportation, women′s and husbands′ lower education levels, lack of mass media exposure, women′s low autonomy, and shortage of healthcare providers (LHWs). These findings are consistent with the previous research carried out in multiple countries, including Nigeria [48], Ghana [49], Mali [50], Zambia [51], Ethiopia [52, 53], India [39], and Nepal [29]. All in all, women′s socioeconomic background is very critical in maternal healthcare utilization. Predominantly, women from poor segments of society with limited resources and autonomy can barely afford out‐of‐pocket expenditures for healthcare [27], restricting them from receiving essential maternal healthcare services, thus instigating inequality in society. Arguably, women with improved education and economic status are more capable of seeking and affording healthcare services [4]. Further, the role of mass media is critical in raising awareness, and the availability of one′s own vehicle is more advantageous to avail ANC services [15, 16, 45, 54].

Results also revealed a strong association between adequate ANC utilization and women′s need factors, such as parity, gravidity, and desire for more children. These results are aligned with previous studies [40, 55], especially concerning women with five or more children, a gravidity of five or above, no desire for additional children, and no history of pregnancy loss. Such women were more likely to seek inadequate ANC utilization for themselves [12, 25].

### 5.1. Policy Implications

This research recommends several policy implications to address the multifaceted challenges hindering adequate ANC utilization in Pakistan and promoting maternal and child healthcare outcomes. This research identified wealth inequality, educational disparities, and geographic access gaps as the strongest and most consistent drivers of inadequate ANC utilization across three decades in Pakistan and, therefore, requires the highest policy priority for improving maternal healthcare coverage. This research is beneficial for policymakers and health managers to implement focused strategies to enhance ANC service utilization within the first three trimesters of pregnant women. This could be achieved through strengthening the existing health facilities and establishing mobile health clinics, particularly in rural and remote areas, to overcome the physical distance that currently excludes the most vulnerable women and improve accessibility to maternal care services, including ANC. These results also recommend launching sustained public health campaigns on a regular basis to raise awareness among pregnant women and their families about the importance of adequate ANC visits, emphasizing the benefits of seeking timely and regular care.

Another critical policy implication advocates for measures to reduce out‐of‐pocket expenditures associated with maternal healthcare services, such as expanding insurance coverage. Strengthening the implementation of initiatives like the Sehat Sahulat Card serves as a pertinent example. Lastly, this research features a policy implication to focus on the efforts for empowering women through education, economic opportunities, and decision‐making autonomy at the household level, all of which can positively influence ANC utilization.

## 6. Conclusion

This research concludes with notable findings regarding the increased utilization of adequate ANC visits in Pakistan over the past three decades. While this upward trend is promising, persistent disparities necessitate immediate attention. Focused interventions, particularly in rural and remote areas, are imperative to increase the uptake of ANC and achieve universal healthcare for mothers and newborns. Addressing service delivery gaps through the establishment of additional healthcare facilities and mobile health clinics for maternal healthcare, especially in marginalized and lower socioeconomic groups, is paramount for ensuring equitable access to ANC. Moreover, expanding the healthcare workforce and ensuring the availability of skilled providers are pivotal for effective ANC services provision and promotion of its benefits.

To further enhance ANC utilization, government efforts should prioritize investment in community awareness campaigns aimed at educating and empowering pregnant women and their families. Initiatives such as transportation infrastructure improvements, facility expansions in underprivileged areas, and financial support are instrumental in bridging healthcare access gaps and ensuring universal access to ANC services. Community outreach programs and health education initiatives play pivotal roles in augmenting awareness about the benefits of ANC, thereby enabling women to make informed healthcare decisions.

## Funding

Open access publishing facilitated by Federation University Australia, as part of the Wiley‐Federation University Australia agreement via the Council of Australasian University Librarians.

## Conflicts of Interest

The authors declare no conflicts of interest.

## Data Availability

Data sharing was not applicable to this article as no datasets were generated or analyzed during the current study.
